# Assessment of Dietary Intake and Nutritional Status in CrossFit-Trained Individuals: A Descriptive Study

**DOI:** 10.3390/ijerph17134772

**Published:** 2020-07-02

**Authors:** Anna Gogojewicz, Ewa Śliwicka, Krzysztof Durkalec-Michalski

**Affiliations:** 1 Department of Food and Nutrition, Poznan University of Physical Education, 61-871 Poznań, Poland; gogojewicz@awf.poznan.pl (A.G.); k.michalski@awf.poznan.pl (K.D.-M.); 2 Department of Physiology and Biochemistry, Poznan University of Physical Education, 61-871 Poznań, Poland; 3 Institute of Human Nutrition and Dietetics, Poznan University of Life Sciences, 61-871 Poznań, Poland

**Keywords:** nutrition, functional training, physical activity, sport, body composition

## Abstract

CrossFit is a discipline with high training and nutritional requirements. To date, there is only scarce data evaluating nutrition among CrossFit training and they mostly focus on selected nutritional interventions. Therefore, the purpose of this descriptive study was the assessment of dietary intake and nutritional status in a selected group of CrossFit-trained participants. The study consisted of 62 CrossFit athletes (31 men and 31 women, aged 31.0 ± 5.2 and 30.0 ± 4.3 years, respectively). Body composition was analyzed by electrical bioimpedance. Dietary intake was assessed using a standardized 3-day food record. Body fat percentage for females and males was 20.3 ± 4.3% and 13.7 ± 3.3% respectively. The energy intake in the diet was lower (~1700 kcal in women and ~2300 kcal in men) than the recommended demand. Moreover, low consumption of carbohydrates was stated, as well as an inadequate intake of folate, vitamin E (in women), and minerals, such as Fe and Ca (in women). The energy, carbohydrate, iron, and calcium intake in the CrossFit participants’ diet was too low in comparison to recommendations. It seems justified to educate athletes and coaches about nutritional habits, and individual energy and nutrients requirements.

## 1. Introduction

In recent years, CrossFit training has become very popular sport discipline. According to Glassman [1], who is the founder of CrossFit, the goal of CrossFit training is to improve ten general physical skills: cardiovascular/respiratory endurance, stamina, strength, flexibility, power, speed, coordination, agility, balance, and accuracy. It is recognized as a high-intensity functional training program which combines many types of exercise, e.g., Olympic weightlifting, powerlifting, sprints, plyometrics, calisthenics, gymnastics, and running. These exercises are usually combined into high-intensity workouts called “workout of the day” (WOD), that are performed quickly, repetitively, with limited or no recovery time between sets [2,3]. The workouts are scalable and take into account the current fitness level of the person, so that exercises can be performed effectively and safely [2].

The effectiveness of sports training depends on a properly planned training program and a well-balanced diet. Providing an inadequate energy and nutrient intake can reduce the body’s adaptation to physical activity. It also leads to a decrease in lean body mass, muscle strength and endurance, decreased immunity, and health complications [4]. CrossFit is very often associated with strict dietary behaviors. According to the recommendations of the CrossFit founders, high amounts of protein, up to 30% of energy intake, should be consumed. Likewise, the intake of fat in the diet should cover 30% of the daily energy requirements (mainly in the form of mono- and polyunsaturated fatty acids). The contribution of carbohydrates in the diet was originally recommended at a low level (about 40% of the daily energy requirements) [2]. Furthermore, CrossFit trainers most frequently recommend the Paleo and Zone diets [5]. According to Glassman [6], such a share of macronutrients in these diets is necessary for permanent weight loss and optimal health. It should be underlined that the aforementioned nutrition strategies are controversial because they do not meet the recommendations of esteemed associations such as International Society of Sports Nutrition (ISSN) [4]. Considering the effectiveness of exercise performance in CrossFit training, nutritional status, and the health of athletes, it seems reasonable to implement in practice dietary recommendations that are consistent with the principles of rational nutrition.

However, we would like to underline that there is only scarce data evaluating nutrition among CrossFit training [7] and they likely focused on selected nutritional interventions e.g., ketogenic diet [3,8], pre- and post-workout ingestion of proteins and carbohydrates [9,10,11,12,13], or use of some ergogenic supplements [14,15,16,17]. Therefore, the purpose of this descriptive study was the assessment of customary dietary intake and nutritional status in a selected group of CrossFit-trained participants and in natural training conditions.

## 2. Materials and Methods

### 2.1. Study Group

The study group consisted of 31 men and 31 women (age 31.0 ± 5.4 and 30.0 ± 4.1 years, respectively). All subjects were free of injury and known illness and had been participating in at least three CrossFit workouts per week for at least six months. They were at a similar moderate athletic level and practicing CrossFit for about two years. The characteristics of the participants are given in Table 1. All subjects declared that they had not introduced any changes in their lifestyles, elements of training, nutrition, and/or supplementation. The study protocol was reviewed and approved by the Bioethics Committee at Poznań University of Medical Sciences, reference numbers 681/16 and 683/16 (10 November 2016). All participants in the study gave their written informed consent. All procedures were conducted in accordance with the ethical standards of the 1975 Helsinki Declaration.

### 2.2. Anthropometric and Body Composition Measurements

All anthropometric measurements were conducted in the laboratory, in a fasting state and in the morning hours by the same specialist (certified dietitian). Body mass and height were measured using a certified digital medical-grade scale and a mechanical measuring rod (WPT 60/150.O, Radwag, Radom, Poland) with accuracy of 0.1 kg for weight and 0.5 cm for height, respectively. Body composition was assessed by the bioimpedance method, using the BIA 101S analyzer and Bodygram 1.31 computer software (AKERN-RJL, Pontassieve, Italy). Body composition was measured strictly following the recommended measurement conditions as described previously [18].

### 2.3. Daily Energy Expenditure

Assessment of total daily energy expenditure (EE) was conducted according to a previously validated method [19], in which heart rate (HR) monitoring data (Polar RS-400, Vantaa, Finland) were used. Each participant’s HR was recorded minute-by-minute for five consecutive days, including weekends. They were also asked to report time and type of habitual daily activities and training, as well as information that was important for elimination of any accidental errors (e.g., cell phone interference, loss of skin contact during sleep) in HR recordings. The information was used to fill the potential gaps in HR recordings. The obtained wrist-worn HR data were downloaded to a computer equipped with the Polar ProTrainer 5 software (version 5.41.002, Polar, Vantaa, Finland). On a separate visit, the thresholds HR (HR_FLEX_) for activity categories (sedentary, light, moderate, and vigorous) were estimated individually for each participant. Energy expenditure (EE) was calculated for each category according to recommendations and recorded five-day HR data were categorized to the intensity levels and used to estimate total daily energy expenditure [19,20].

### 2.4. Nutritional Assessment

The assessment of dietary intake was conducted using a standardized 3-day food record. The participants were asked for expression of the amount of meals in common measurement units (e.g., glass, cup, bowls, spoons, etc.). The obtained information was adjusted using the album of photographs of food products and dishes elaborated by The National Food and Nutritional Institute in Warsaw. As in our previous works [3,21,22], quantitative analysis of the composition of the daily food rations was performed using the Dietetyk 2016 (Jumar, Poznań, Poland) software package. This program is based on a database elaborated by The National Food and Nutritional Institute. Meanintakes of energy and nutrients were compared with recommendations of the ISSN [4].

### 2.5. Statistical Analysis

This is a descriptive study, focusing only on one time point nutritional assessment and anthropometric measurements in CrossFit-trained individuals. Data are presented as means, standard deviations (SD), medians (Me), and quartiles (Q1–Q3). The Shapiro–Wilk test was used to check the data for normal distribution. The level of statistical significance was set at *p* < 0.05. The Pearson analysis for normally distributed variables and Spearman’s rank analysis for non-normally distributed variables were used to calculate correlation coefficients. All analyses were performed using the Statistica 13.0 software package (StatSoft, Tulsa, OK, USA). Before the study, the sample size evaluation indicated that 45 subjects were needed assuming a medium effect size, and according to α-level of 0.05 (G*Power; Heinrich-Heine-Universität, Düsseldorf, Germany).

## 3. Results

### 3.1. Anthropometry and Body Composition

The body composition analysis of CrossFit-trained participants showed normative values for all studied CrossFitters (Table 1).

### 3.2. Nutritional Evaluation

Table 2 presents the average energy value of diet and selected nutrients intake. The energy intake of athletes’ diet amounted to about 1736 kcal in women and 2265 kcal in men, while the daily total EE was 2598 kcal and 2828 kcal, respectively. The energy availability was below the ISSN recommendations [4]. Average protein intake met the recommended value and oscillated at about 1.6 g/kg of body mass (about 92 g in women and about 135 g in men, respectively). According to ISSN recommendations, the carbohydrate intake should be within the range of 5–8 g of body mass per day for physically active people [4]. However, in our study, carbohydrate intake was lower (~3.9 g/kg of body mass in women and ~3.3 g/kg of body mass in men) than the reference values. Moreover, the share of energy derived from fats in both groups was normative and did not exceed the maximum value of 30%. According to European Food Safety Authority (EFSA) [23] the average amount of cholesterol consumed in both women’s and men’s diets exceeded the reference values of 346 mg and 479 mg, respectively.

Table 3 presents the average nutritional value of selected vitamins and minerals. In men and woman, the correct vitamins intake was observed according to the recommendations or slightly exceeding it; only vitamin E intake in women was too low. Average sodium (~1331 mg for women and ~1856 mg for men) and potassium intake (~3637 mg for women and ~5250 mg for men) in all the studied athletes seemed to be rational compared to the recommendations. The average calcium intake in women’s diet was low (~894 mg) and optimal in men (~1214 mg) with regards to recommendations [4]. The women’s diet was characterized by a lower iron, zinc, and folic acid intake as well as higher phosphorus and magnesium intake in comparison to the reference values. However, in males, the intake of these components was slightly higher than the recommended standards [4].

**Figure 1:**
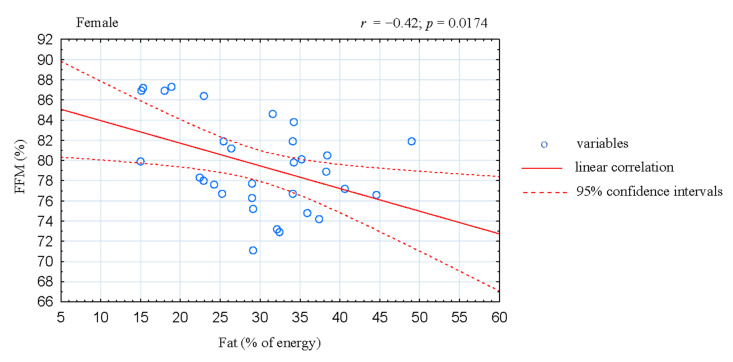
Correlation between dietary fat intake and fat-free mass (FFM) in female CrossFit-trained individuals.

**Figure 2:**
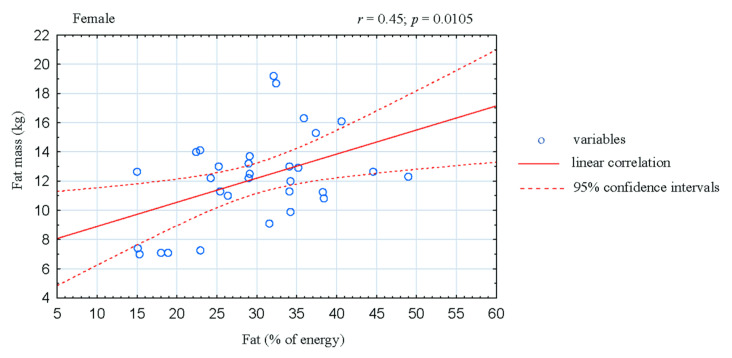
Correlation between dietary fat intake and body fat mass in female CrossFit-trained individuals.

**Figure 3:**
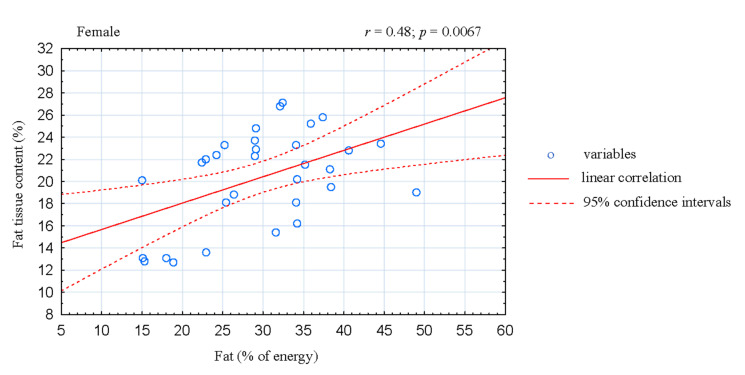
Correlation between dietary fat intake and fat tissue content in female CrossFit-trained individuals.

**Figure 4:**
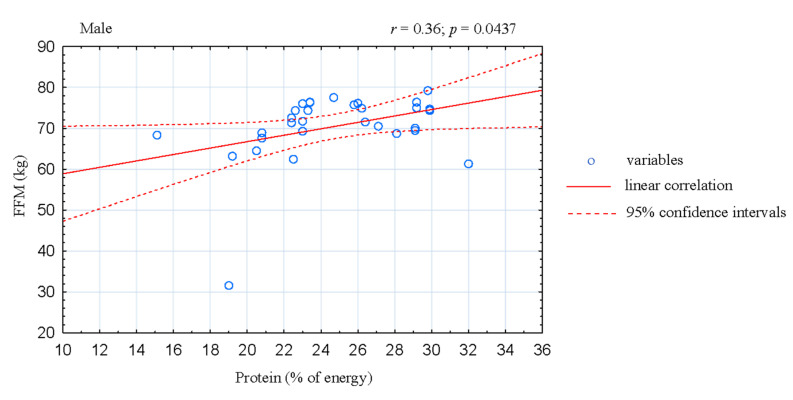
Correlation between dietary protein intake and FFM in male CrossFit-trained individuals.

The analysis of correlation in the women CrossFitters showed negative association between the share of energy derived from fats and fat-free mass (FFM; *r* = −0.42; *p* = 0.0174; Figure 1). Moreover, the share of energy derived from fats was positive correlated with body fat mass (*r* = 0.45; *p* = 0.0105; Figure 2) and fat tissue content (*r* = 0.48; *p* = 0.0067; Figure 3). In the male athletes we found positive correlations between the share of energy derived from proteins and FFM content (*r* = 0.36; *p* = 0.0437; Figure 4).

## 4. Discussion

CrossFit is a discipline with high training and nutritional requirements. To the best of our knowledge, this is the first study on the nutrition and nutritional status evaluation of moderately trained CrossFit practitioners. Body composition of studied CrossFitters was similar with the reports of other authors [25,26,27].

The assessment of the diet among enrolled CrossFitters showed that the energy intake was lower than the daily energy demand (Table 2). The phenomenon of low energy availability among athletes is very common across what was observed in the current study and among others in long-distance runners [28,29], cyclists [30,31], and in competitions where weight categories apply, e.g., martial arts, rowing [32,33]. Our study conducted in a group of currently popular discipline (CrossFit) are innovative and provide the current scientific gap in this respect. It should also be emphasized that chronic energy deficiency and/or high energy expenditure related to physical activity may lead women to the menstrual disorders and low bone mineral density, as well as weight loss, dehydration, excessive fatigue, and gastrointestinal and other health-related problems [34,35]. Similarly, in male athletes, low energy availability leads to a number of health problems [36], including low testosterone [37] or insulin and leptin concentrations irregularities [38].

Proper assessment and calculation of daily energy intake is an important element in the discussion on low energy availability. Numerous works indicate intentional or unintentional errors in estimating portion sizes, omitting snacks, drinks, or reduced food intake during the study period [39]. However, the studied CrossFitters were characterized by normal body weight, so it can be assumed that the energy intake among the studied athletes may have been sufficient. In addition to the energy content of the diet, the nutrient intake in the athlete’s diet is also important. Firstly, in this regard, adequate protein intake in the diet is crucial to muscle adaptive response to exercise training, especially for muscle protein synthesis stimulation, protein breakdown suppression, recovery mechanisms after exercises, as well as maintain proper functioning of the body and final training adaptation and/or competition results [40]. The intake of protein, both in the group of studied women athletes and in the group of male athletes per kg of body weight, turned out to be sufficient. It is worth noting that the studied athletes consumed high quality protein, mainly of animal origin, beneficial in the diet of the athlete. All athletes provided at least 1.5 g of protein/kg of body weight from food. This is in accordance with ISSN [4] recommendations, according to which, it amounts to 1.4–2.0 g/kg of body weight for strength and endurance disciplines, including CrossFit. Moreover, the positive correlation found in our study between the share of energy derived from proteins and FFM content in male athletes (Figure 3) showed that protein intake was sufficient. However, given the latest data of Morton et al. [41], this intake in our opinion could not be sufficient to maximize the possibility of stimulating hypertrophy and increasing muscle strength/power (1.6–2.2 g/kg).

Secondly, carbohydrates are the main source of energy in athletes’ diets. CrossFit training is highly intensive and requires an adequate intake of carbohydrates in order to build up muscle glycogen reserves and thus provide the necessary energy for muscle work [12]. In our study, the CrossFitters consumed too low amounts of carbohydrates (Table 2) compared to ISSN recommendations (5–8 g/kg per day). Moreover, we would like to underline that there are currently no clear guidelines on the amount of carbohydrates and their impact on the ability to CrossFit-specific training. Pendergast et al. [42] suggest that during intensive anaerobic training the carbohydrate intake should be 8–10 g/kg/day or 60–70% of energy intake. Burke et al. [43] recommends carbohydrates intake by adults in the range of 5 to 7 g/kg/day for moderate activity for about 1 h a day and 6 to 10 g/kg/day for moderate to high intensity exercise for 1–3 h of training a day. It should also be noted that proper carbohydrate intake has a significant influence on exercise performance and physical capacity [21,44]. Irregularities in consumption of this macronutrients increases the risk of injury in athletes [45] and contributes to the deterioration of training/competition results [21,46]. In addition, a low carbohydrate intake combined with a low energy availability may result in the use of amino acids (derived from structural protein) as an additional and alternative energy substrate [43].

Thirdly, fat consumption in the studied athletes did not exceed recommended intake (~30%) [4]. However, a large discrepancy was observed in the quality and quantity of fat consumed, both in women and men (Table 2). In addition, in women athletes the share of energy derived from fats was negatively associated with FFM (Figure 1) and positively correlated with body fat mass and fat content (Figure 2 and Figure 3). In accordance with the recommendations for maintaining health, the value of 35% of energy derived from fat should not be exceeded; excessive fat consumption may also negatively affects sports performance [4,47]. Furthermore, most of the diets analyzed in our study were rich in cholesterol. This can be explained by the high intake of food of animal origin containing cholesterol and saturated fatty acids, especially eggs and meat products, which has also been observed by other authors [48].

Fourthly, the proper nutritional intake of vitamins and minerals is also crucial in athletes. Physical exercise induces increased generation of reactive oxygen species (ROS) involved in muscle damage, immune dysfunction, and fatigue [49]. Research results suggest that the oxidative stress response is proportional to the intensity of physical activity [50]. However, it should be remembered that ROS not only cause damage to muscle fibers but can also play a role in cellular signaling in the process of adaptation to training which is a positive phenomenon [51]. However, the demand for vitamins and minerals may be simultaneously growing in athletes and physically active people. On the other hand, the widespread use of vitamin supplementation (e.g., vitamin E and C) among athletes is still controversial. McLeay et al. [52] suggest that continuous supplementation weakens the ability to adapt to exercise. In our study, vitamin C intake was sufficient in most athletes, while vitamin E intake in female athletes was below recommendation. Our results are in line with those obtained by other authors [53]. According to Reboul [54], low dietary intake of vitamin E is common in both Europe and the US populations. Moreover, it may be the result of low stability of vitamin E in vegetable oils [54]. This is important because vitamin C and vitamin E supplementation reduces oxidative stress and can only have a beneficial effect in athletes with a low initial concentration of this ingredient [55]. Furthermore, the intake of vitamin A in the studied athletes was slightly higher than recommended. Negative consequences of accumulation of this component may lead to excessive excitability and coordination disorders. However, in the case of a natural intake of this vitamin in food, any risk seems to be insignificant.

A significant folic acid deficiency was observed in the women’s diet (Table 3). Inadequate intake of this component was also observed by Chryssanthopoulos et al. [53] in footballers. Folic acid deficiency may be caused by low energy content of the diet. However, in women the deficiency of folates may lead to the development of anemia together with dysfunction of the central nervous system [56]. It is known that regular activity increases bone mineral density [57] and phosphorus, calcium and magnesium are essential in the construction of their proper structure [58]. These components also determine the proper neuromuscular excitability and muscle contraction capacity [59]. Food rations of all the studied athletes contained much higher amounts of magnesium and phosphorus (Table 3). However, a serious nutritional problem was registered in a too low calcium intake in the studied women. For men, the intake of this element with the diet covered the total daily requirement, which was set for both sexes at above 1000 mg/day. Intake of vitamin D in all the athletes was at an appropriate level (Table 3). It is well known that low calcium intake associated with vitamin D deficiency may lead to deterioration of bone mineralization and to fractures. Therefore, it is recommended to maintain optimal vitamin D, calcium, and phosphorus intake in athletes’ diet [60] due to the fact that e.g., vitamin D can affect workouts and sports results. What is more, the nutritional status of vitamin D in athletes is influenced by the type of activity, season, and geographical location differences. Therefore, the potential risk of vitamin D deficiency at any time of year exists in CrossFit athletes, who exercise and compete indoors, avoiding exposure to the sun.

The studied athletes revealed normative sodium intake (Table 3). Teshima et al. [61] analyzed the diet of Japanese karate athletes and showed a similar trend. However, according to Benardot [62], in cases of significant loss of sodium with sweat, the recommended intake for this ingredient can be as high as 10 g per day. Sodium, specifically, is the major ion of the extracellular fluid, and has several important functions during exercise including fluid retention [63]. Simultaneously, the studied group was characterized by desirable dietary intake of potassium (Table 3). Teshima et al. [61] obtained completely opposite results among combat sports athletes, in whom low potassium intake was explained by low consumption of fruit and vegetables. From a practical point of view, these observations can be seen as significant because electrolyte (Na^+^ and K^+^) concentration imbalance may lead to painful, sudden, and involuntary skeletal muscle cramp during or after training [64].

In terms of mineral intake, we also wanted to point out that average iron intake in the diet of the studied female CrossFitters was slightly below the recommended value (Table 3). Reduced iron levels can cause a decrease in exercise capacity and a deterioration in athletic performance [65]. Furthermore, Iglesias-Gutierrez et al. [66] have shown in their studies that reduced iron levels can occur even if the intake of this ingredient from the diet corresponds to the recommended daily intake. Therefore, regular monitoring of this ingredient, a diet rich in iron or a possible supplementation seems to be justified.

Finally, it is worth mentioning that the creators of CrossFit most often recommend Paleo and Zone diets. However, as shown by Maxwell et al. [5] the nutritional knowledge of certified CrossFit instructors is insufficient and indicates the need to educate athletes and coaches on nutritional needs and individual energy requirements.

This study has some limitation. It cannot be ruled out that a too low diet’s energy intake may be due to underestimation and/or inaccurate recording of the products consumed by athletes [67]. However, the authors of the study tried to minimize the risk of this fact by educating the athletes in the aspect of proper diet recording and constant contact with them in this regard. In addition, extended interpretation of long-term nutritional disparities requires further consideration of various nutrient–nutrient interactions as well as food interactions on bioavailability, supported by complementary data on actual nutritional status at baseline as well as during and after the study. Moreover, in our study participated only moderately trained CrossFit contestants, hence potential results extrapolation to elite CrossFit competitors should be treated with caution.

## 5. Conclusions

In conclusion, in our study the diet of the CrossFit-trained participants indicates a risk of nutritional irregularities, which could lead to nutritional status and performance disturbances over longer periods of time. The energy, carbohydrate, iron, and calcium content in the CrossFitter’s diet reveals lower intake compared to recommendations. Therefore, CrossFitters have to be encouraged to support their diet with nutrient-dense whole and fresh products such as whole grains, dairy products, legumes, fruit, and vegetables. Based on the data collected, it is suggested that more research is needed in order to obtain unambiguous information on nutrition in CrossFit. It seems to be also justified to educate athletes and coaches about nutritional habits, and individual energy and nutrients requirements.

## Figures and Tables

**Table 1:** Anthropometric characteristics of studied CrossFit-trained participants.

Variable	Unit	Women (*n* = 31)	Men (*n* = 31)
Age	years	30.0 ± 4.1	31.0 ± 5.4
		29.0 (28.0–34.0)	29.0 (27.0–35.0)
Body height	m	1.67 ± 0.06	1.78 ± 0.06
		1.68 (1.62–1.71)	1.78 (1.75–1.82)
Body mass	kg	59.3 ± 5.2	82.5 ± 7.4
		58.6 (54.9–62.8)	81.9 (78.2–87.5)
BMI	kg/m^2^	21.3 ± 1.9	26.0 ± 1.9
		20.9 (20.0–22.0)	25.7 (25.1–27.2)
FAT	%	20.3 ± 4.3	13.7 ± 3.3
		21.5 (18.1–23.3)	14.1 (11.2–15.8)
	kg	12.1 ± 3.1	11.4 ± 3.4
		12.3 (10.8–13.7)	11.1 (8.6–13.4)
FFM	%	79.5 ± 4.5	86.3 ± 3.3
		78.9 (76.6–81.9)	85.9 (84.2–88.8)
	kg	47.2 ± 3.9	70.5 ± 8.6
		47.9 (42.7–50.3)	71.7 (68.7–75.7)
TBW	%	57.6 ± 3.7	62.0 ± 3.0
		56.5 (55.1–59.4)	61.7 (60.3–63.9)
	L	33.8 ± 2.9	50.8 ± 4.0
		33.2 (31.5–35.6)	51.6 (48.6–53.6)

Values are expressed as means ± standard deviations (SD), medians (Me) and quartiles (Q1–Q3). BMI, body mass index; FAT, fat mass; FFM, fat-free mass; TBW, total body water content.

**Table 2:** Mean values of energy and macronutrients intake in CrossFit athletes.

Variable	Unit	Women (*n* = 31)	Men (*n* = 31)	ISSN Recommendations [4]
Energy Expenditure	kcal	2598 ± 286	2828 ± 316	
		2708 (1982–3022)	2910 (2236–3327)	
Energy Intake	kcal	1736 ± 407	2265 ± 417	600–1200/h during exercise 2500–8000 kcal/day
		1736 (1401–2072)	2150 (1924–2425)	
	kcal/kg	29.5 ± 7.6	27.8 ± 6.3	50–80 kcal/kg/day
		28.2 (25.1–33.7)	24.9 (22.8–32.4)	
Carbohydrate	% energy	48.0 ± 8.6	44.8 ± 10.1	55–60%
		49.3 (43.5–54.4)	43.2 (35.0–51.8)	
	g	229 ± 72	273 ± 100	250–1200g/day
		219 (187–261)	255 (210–332)	50–150 kg athlete
	g/kg	3.9 ± 1.3	3.3 ± 1.3	5–8 g/kg/day
		3.7 (3.4–4.3)	3.0 (2.4–3.8)	
Dietary Fiber	g	29.0 ± 11.8	34.6 ± 9.5	25 [24]
		23.9 (19.0–37.2)	33.0 (26.7–42.3)	
Protein	% energy	22.0 ± 6.0	24.7 ± 4.0	
		21.7 (18.2–24.8)	23.4 (22.4–29.1)	
	g	92 ± 24	135 ± 30	75–300 g
		89 (76–109)	133 (116–150)	
	g/kg	1.6 ± 0.4	1.6 ± 0.4	1.4–2.0 g/kg/day
		1.5 (1.3–1.7)	1.6 (1.4–1.8)	
Fat	% energy	29.7 ± 8.6	30.5 ± 9.8	30%
		29.1 (22.9–35.2)	28.0 (23.1–37.8)	
	g	61 ± 24	80 ± 22	
		63 (46–76)	81 (60–89)	
	g/kg	1.0 ± 0.4	1.0 ± 0.3	0.5–1 g/kg/day
		1.0 (0.8–1.3)	0.9 (0.7–1.2)	
SFA	g	19.6 ± 8.7	22.1 ± 6.6	
		17.1 (12.1–26.5)	21.8 (17.2–28.6)	
MUFA	g	20.8 ± 6.8	29.6 ± 8.8	
		20.6 (17.1–25.4)	28.3 (23.5–35.6)	
PUFA	g	12.4 ± 5.0	14.5 ± 4.2	
		12.8 (8.7–14.9)	15.8 (11.9–16.5)	
Cholesterol	mg	346 ± 522	479 ± 286	
		235 (92–381)	490 (206–676)	

Values are expressed as means ± SD and Me (Q1–Q3). ISSN, International Society of Sports Nutrition; SFA, saturated fatty acids; MUFA, monounsaturated fatty acids; PUFA, polyunsaturated fatty acids.

**Table 3:** Mean values of vitamins and minerals intake in CrossFit athletes.

Vitamin/Mineral	Unit	Women (*n* = 31)	Men (*n* = 31)	ISSN Recommendations [4]	
				Women	Men
Vitamin A	µg	1184 ± 785	1651 ± 1032	700 µg/d	900 µg/d
		886 (628–1826)	1226 (1006–2564)		
Vitamin D	µg	5.8 ± 6.2	6.3 ± 5.8	5 µg/d	
		2.0 (0.8–10)	5.0 (2.9–9.0)		
Vitamin C	mg	110 ± 104	172 ± 210	75 mg/d	90 mg/d
		84.8 (50.9–121)	133 (98.6–206)		
Vitamin E	mg	12.7 ± 9.4	16.9 ± 8.5	15 mg/d	
		9.0 (7.7–15.0)	15.0 (11.6–20.7)		
Vitamin B_1_	mg	1.1 ± 0.6	1.4 ± 0.5	1.1 mg/d	1.2 mg/d
(Thiamin)		1.1 (0.8–1.3)	1.4 (0.9–2.0)		
Vitamin B_2_	mg	1.9 ± 0.7	2.7 ± 1.2	1.7 mg/d	1.3 mg/d
(Riboflavin)		1.8 (1.4–2.5)	3.1 (2.0–3.5)		
Vitamin B_3_	mg	21.4 ± 11.6	30.3 ± 9.6	14 mg/d	16 mg/d
(Niacin)		19.5 (13.8–24.0)	28.7 (21.3–37.0)		
Vitamin B_6_	mg	2.7 ± 0.8	3.7 ± 1.3	1.3 mg/d	
(Pyridoxine)		2.7 (2.1–3.0)	3.7 (2.8–4.4)		
Vitamin B_12_	µg	3.6 ± 3.3	5.0 ± 4.1	2.4 µg/d	
(Cyanocobalamin)		3.1 (0.8–45)	3.9 (0.8–7.3)		
Folic acid	µg	289 ± 142	375 ± 152	400 µg/d	
		253 (203–314)	337 (274–520)		
Sodium	mg	1331 ± 591	1856 ± 979	500 mg/d	
		1036 (945–1914)	1565 (1182–2553)		
Potassium	mg	3637 ± 1083	5250 ± 1630	2000 mg/d	
		3348 (2947–4634)	5512 (4058–5991)		
Calcium	mg	894 ± 431	1214 ± 550	1000 mg/d	
		871 (530–1304)	1121 (786–1398)		
Phosphorus	mg	1632 ± 497	2277 ± 707	700 mg/d	
		1541 (1362–1914)	2236 (1757–2726)		
Magnesium	mg	407 ± 96.2	552 ± 177	320 mg/d	420 mg/d
		371 (339–463)	536 (462–653)		
Iron	mg	12.6 ± 3.2	16.5 ± 3.6	18 mg/d	8 mg/d
		11.9 (11–14)	15.6 (14–18.3)		
Zinc	mg	10.7 ± 2.7	14.7 ± 4.25	8 mg/d	11 mg/d
		10.5 (8.8–11.7)	13.7 (11.3–18)		

Values are expressed as means ± SD and Me (Q1–Q3).
